# Curcumin-Loaded Nanoscale Metal–Organic Frameworks for Therapeutic Applications in Cancer

**DOI:** 10.3390/nano15241869

**Published:** 2025-12-12

**Authors:** Helda Tutunchi, Hafezeh Nabipour, Sohrab Rohani

**Affiliations:** 1 Endocrine Research Center, Tabriz University of Medical Sciences, Tabriz 51666-14711, Iran; 2 Department of Chemical and Biochemical Engineering, University of Western Ontario, London, ON N6A 5B9, Canada

**Keywords:** cancer therapy, curcumin, drug delivery systems, metal–organic frameworks

## Abstract

Curcumin is a naturally occurring polyphenol that has gained attention in cancer research due to its anti-inflammatory, antioxidant, and anticancer properties. However, its clinical use is limited due to poor water solubility, rapid degradation, and low bioavailability, which reduce its therapeutic effectiveness. To overcome these issues, curcumin has been combined with other agents, including chemotherapeutic drugs, photothermal materials, and metal-based compounds, to improve stability and antitumor activity. Biocompatible drug-delivery systems that allow controlled or sustained release are particularly valuable in oncology, as they can minimize side effects and improve treatment efficiency. Among these carriers, metal–organic frameworks (MOFs) have emerged as promising platforms due to their porous structure, tunable chemistry, and high loading capacity. This review focuses on the potential of MOFs as nanocarriers for curcumin, emphasizing their ability to enhance stability, increase bioavailability, improve therapeutic outcomes, and deliver the drug selectively to tumor sites.

## 1. Introduction

Noncommunicable diseases (NCDs) represent one of the most pressing global health challenges, responsible for approximately 74% of all deaths worldwide [1]. Among these conditions, cancer remains a dominant threat. In 2022 alone, the World Health Organization reported nearly 20 million newly diagnosed cases along with more than 9 million cancer-related deaths. Without substantial progress in preventive strategies and therapeutic options, the global incidence of cancer is projected to rise steeply, potentially reaching 35 million new cases annually by 2050. Such alarming trends highlight the urgent necessity for innovative diagnostic tools and treatment approaches that can complement existing clinical routines to ensure earlier detection, more precise interventions, and improved patient outcomes [2,3]. Although notable advancements have recently been achieved in cancer research, conventional treatments—including surgery, chemotherapy, and radiotherapy—still face important limitations. A major issue with systemic therapies is their lack of specificity; while anticancer agents aim to eliminate malignant cells, they inevitably harm normal tissues as well, leading to severe side effects that restrict treatment dosage and reduce patients’ quality of life. Moreover, many tumors possess inherent resistance mechanisms or acquire resistance during therapy, ultimately diminishing long-term treatment efficacy and contributing to higher recurrence rates. Another critical challenge lies in the inability to tailor therapeutic regimens dynamically based on real-time patient responses, which significantly constrains the development of truly personalized cancer care [4,5,6].

A growing body of research indicates that natural compounds—especially those sourced from medicinal plants—are playing an increasingly significant role in both the prevention and management of cancer [7,8,9,10]. Among these bioactive substances, curcumin stands out as a hydrophobic polyphenolic constituent extracted from the rhizomes of *Curcuma longa* L. (turmeric). This molecule exhibits a wide spectrum of biological activities and has been recognized for its anti-inflammatory, antioxidant, antimicrobial, and anticancer capabilities [11,12,13,14,15,16].

A substantial body of research has shown that curcumin and its nanoscale derivatives exhibit therapeutic promise across a wide range of cancer types [15,16]. Curcumin exerts its biological effects in part by suppressing the production of pro-inflammatory mediators and safeguarding cartilage matrix components from degradation, a process largely mediated through inhibition of the nuclear factor kappa B (NF-κB) and mitogen-activated protein kinase (MAPK) signaling pathways. In addition, curcumin mitigates oxidative stress by neutralizing reactive oxygen species (ROS), enhancing the activity of endogenous antioxidant enzymes, and reducing chondrocyte apoptosis through downregulation of cleaved caspase-3 and Bax while promoting Bcl-2 expression [17,18,19]. Curcumin has also been shown to potentiate the antitumor efficacy of chemotherapeutic agents such as cisplatin and 5-fluorouracil, while simultaneously diminishing their associated toxicities through synergistic pharmacological interactions [20]. Growing preclinical evidence further indicates that curcumin modulates several key molecular pathways involved in tumor initiation and progression—including NF-κB, signal transducer and activator of transcription 3 (STAT3), and the phosphoinositide 3-kinase/protein kinase B (PI3K/Akt) axis—thereby inhibiting cancer growth and promoting apoptosis in numerous experimental models [21,22,23,24]. Despite these multifaceted anticancer activities, curcumin’s translation into clinical practice remains constrained by its unfavorable biopharmaceutical characteristics [25]. Its poor aqueous solubility, limited intestinal absorption, rapid metabolic conversion, and swift systemic clearance collectively result in extremely low bioavailability and therefore suboptimal therapeutic performance. These challenges have prompted intensive research efforts aimed at enhancing curcumin’s delivery efficiency, stability, and tissue targeting [25,26]. Among the various strategies explored, the encapsulation of curcumin within drug-delivery systems (DDSs) has emerged as one of the most effective approaches to overcome these limitations and strengthen its clinical applicability [27,28].

DDSs refer to specialized formulations or devices designed to administer therapeutic agents with enhanced safety and efficacy by controlling the rate, timing, and location of drug release [29]. Beyond enabling regulated drug delivery, these systems also aim to improve patient adherence, minimize off-target effects, and direct therapeutics toward specific cellular receptors. To achieve such functions, delivery platforms are constructed from various combinations of organic and inorganic materials, with the overarching goal of improving drug-transport efficiency and therapeutic outcomes. Examples of these platforms include liposomes, polymeric micelles, layered double hydroxides, mesoporous silica nanoparticles, quantum dots, transition-metal dichalcogenides, and covalent organic frameworks [30,31,32,33,34,35,36,37]. Although inorganic carriers can suffer from concerns related to toxicity and limited biodegradability, many organic carriers often exhibit insufficient drug-loading capacity, restricting the amount of therapeutic agent that can be incorporated [38]. Over the past two decades, metal–organic frameworks (MOFs) have emerged as a distinct class of hybrid crystalline materials composed of inorganic metal nodes interconnected by organic linkers [39]. Owing to their structural versatility, MOFs have gained significant attention across multiple application fields—including gas storage, molecular separation, catalysis, purification, energy systems, and environmental remediation. Compared with conventional delivery materials, MOFs possess several distinctive characteristics that make them particularly attractive as DDS components. Their inherently high porosity, large pore volumes, and exceptionally high surface areas allow for efficient encapsulation of drug molecules or other guest species. Furthermore, attributes such as facile functionalization, biocompatibility, tunable water dispersibility, and potential biodegradability contribute to improved bioavailability and therapeutic performance. Drug incorporation into MOFs can occur through chemical attachment or physical entrapment and is governed by various intermolecular interactions, including hydrogen bonding, van der Waals forces, π–π stacking, electrostatic attractions, coordination interactions, and covalent bonding [40,41,42].

Several strategies have been developed to incorporate biomolecules into MOFs, including bio-linking approaches, in situ encapsulation during framework synthesis, post-synthetic pore entrapment, surface attachment, and the creation of intrinsically bio-derived MOFs [38,43]. Owing to their remarkable capacity for hosting guest species, MOFs offer a versatile and powerful platform for drug-delivery applications [38]. Despite these advantages, the in vivo performance of MOF-based systems can be hindered by insufficient accumulation at tumor sites and restricted drug release. To overcome these limitations, a wide range of stimuli-responsive release mechanisms has been investigated, involving triggers such as pH variations, ionic species, ATP, thermal inputs, light, and numerous other combinations [38,44]. Lawson and co-workers evaluated the M-MOF-74 series (where M = Mg, Zn, Co, or Ni) as carriers for curcumin and demonstrated that frameworks constructed from more soluble metal centers exhibited faster curcumin-release profiles [45]. In another study, curcumin was efficiently loaded into an aluminum fumarate MOF (AlMOF) using a simple wet-impregnation technique. This encapsulation approach significantly enhanced the stability of curcumin, resulting in a decreased degradation rate. Moreover, AlMOF incorporation markedly improved curcumin’s antioxidant performance, increasing its activity by nearly threefold [46].

Curcumin-loaded MOFs have been explored as promising platforms for cancer therapy. For instance, Nabipour et al. [47] developed a bio-Schiff base MOF derived from vanillin to serve as a curcumin carrier, achieving an entrapment efficiency of 84.35%. The system was further modified with a carboxymethylcellulose coating, rendering it responsive to pH changes and enabling controlled curcumin release under simulated physiological conditions. Cytotoxicity assessments confirmed its potent anticancer activity. Similarly, Alavijeh et al. [48] demonstrated that encapsulating curcumin within a nanoporous MOF improved its aqueous solubility and markedly increased its cytotoxic effects against human gastric cancer cells relative to unencapsulated curcumin.

This review represents the first comprehensive and comparative analysis of curcumin-loaded MOFs, encompassing the underlying principles of synthesis, drug-loading strategies, release profiles, and anticancer efficacy across diverse MOF architectures. It emphasizes the development of bio-inspired and targeted MOF platforms, incorporates quantitative assessments of performance improvements, and provides a critical discussion of the obstacles that hinder clinical application. Through this approach, the review offers valuable insights for the rational design and optimization of MOF-based curcumin delivery systems for cancer therapy.

## 2. Fabrication and Characterization of MOFs

The synthesis of MOFs depends on multiple factors, such as reaction time and temperature, the type of solvent, the nature of metal ions and organic linkers, the size and geometry of framework nodes, the presence of counterions, and crystallization kinetics. Together, these parameters affect nucleation and crystal growth. Several synthesis methods are available, each with particular advantages and drawbacks. Frequently used approaches include conventional hydrothermal and solvothermal methods, microfluidic techniques, sonochemical and microwave-assisted synthesis, reverse-phase microemulsions, dry gel conversion, ionothermal and diffusion methods, as well as electrochemical and mechanochemical routes. These diverse strategies offer different reaction conditions and energy inputs, allowing the synthesis to be customized to achieve specific structural and functional properties of the MOF [49,50,51].

A comprehensive assessment of MOFs’ structures, properties, and potential applications necessitates extensive characterization using a variety of analytical techniques to evaluate both physical and chemical attributes. The crystalline architecture is typically examined through powder X-ray diffraction (PXRD) and single-crystal X-ray diffraction (SCXRD). Molecular vibrations are probed using infrared (IR) and Raman spectroscopy, while surface area measurements are generally conducted via the Brunauer–Emmett–Teller (BET) method. Thermal stability is assessed through thermogravimetric analysis (TGA) and differential scanning calorimetry (DSC). For compositional analysis, inductively coupled plasma mass spectrometry (ICP-MS) is employed to quantify elemental content, and X-ray photoelectron spectroscopy (XPS) provides insight into surface chemical states. Nuclear magnetic resonance (NMR) offers detailed information on the structural and dynamic behavior of various nuclear species, whereas scanning electron microscopy (SEM) and transmission electron microscopy (TEM) enable high-resolution imaging of MOF morphology and internal structure [52,53,54,55].

## 3. MOFs for Drug Delivery: Loading and Release

MOFs constitute an innovative class of DDs, notable for their structural versatility and tunable functionalities. Before their therapeutic application, a thorough evaluation of biocompatibility is essential. Ensuring low toxicity and compatibility with biological systems largely depends on the careful selection of metal ions and organic linkers during MOF synthesis. Numerous MOFs utilize essential metals such as iron, zinc, copper, and magnesium, which are naturally present in the human body. Metals, including zinc, iron, magnesium, calcium, and zirconium, are particularly preferred for designing biocompatible MOFs due to their minimal toxicity. Likewise, the choice of organic linkers is critical, as they should be metabolizable or readily excretable to prevent harmful effects and maintain the overall safety of the MOF-based delivery system [56,57]. The inherent limitations of many therapeutic agents, such as poor tissue penetration, chemical instability, and rapid systemic clearance, have driven the exploration of innovative drug-delivery strategies. In this context, MOFs have been engineered as carrier systems to overcome these challenges by facilitating immune evasion, enhancing tumor cell uptake, and boosting the overall efficacy of therapeutics [58]. The large surface area and adjustable porosity of MOFs enable substantial drug-loading capacity, allowing higher concentrations of bioactive compounds to be delivered to target sites. Various strategies can be employed to incorporate drugs into MOFs, leveraging their distinctive structural features. Among these, surface adsorption is considered one of the most effective approaches, owing to the high surface areas and porous architecture of MOFs. Drug incorporation is typically mediated by non-covalent interactions, including van der Waals forces, hydrogen bonding, and electrostatic attractions. These versatile loading mechanisms permit the fabrication of diverse drug-loaded MOFs without being constrained by pore size limitations. Furthermore, the surface properties and chemical functionalization of MOFs are critical in optimizing both drug encapsulation and controlled release, while the characteristics of the solvent used during loading also significantly impact adsorption efficiency, highlighting the importance of careful design in MOF-based DDSs [59].

Despite their considerable potential, nanoscale MOFs face significant challenges in biomedical applications. Many of these frameworks display limited hydrolytic and colloidal stability, which can lead to premature degradation in aqueous or protein-rich biological environments. Additionally, interactions with plasma proteins and other blood components can influence their circulation time, biodistribution, and immunogenic responses. The possible release of metal ions or the presence of non-biodegradable organic linkers further necessitates thorough toxicological evaluation. To address these limitations, strategies such as the incorporation of biocompatible metals, utilization of bio-derived linkers, and surface functionalization—e.g., with polysaccharides or polyethylene glycol (PEG)—have been employed to enhance stability, reduce protein adsorption, and improve overall biocompatibility [56,57].

Nadizadeh et al. [60] incorporated ibuprofen (IBU) into two copper-based MOFs, {Cu_2_(1,4-bdc)_2_(dabco)}_n_ and {Cu_2_(1,4-bdc-NH_2_)_2_(dabco)}_n_ (bdc = benzenedicarboxylic acid, dabco = diazabicyclooctane), via mechanochemical methods, achieving drug-loading efficiencies of roughly 50.54% and 50.27%, respectively. The one-pot loading strategy, where MOF formation and drug encapsulation occur concurrently, has been demonstrated as highly effective. This approach ensures uniform distribution of the therapeutic within the MOF pores, overcoming limitations posed by small pore sizes. Furthermore, the one-pot method improves overall process efficiency by shortening reaction times, minimizing waste, and enabling high drug-loading capacity [61].

For instance, the anticancer agent doxorubicin (DOX) was incorporated into zeolitic imidazolate framework-8 (ZIF-8) via one-pot synthesis, yielding nanocarriers with a drug-loading capacity of 20%, suitable for targeted oncology applications [62]. Another common strategy for drug incorporation is the impregnation method, wherein the MOF is soaked in a solution with a high drug concentration to allow molecular diffusion into its pores. This technique is particularly advantageous for small-molecule therapeutics, which can readily access the internal cavities of the framework, facilitating efficient drug encapsulation [63].

Liu et al. [64] employed an impregnation technique to encapsulate the poorly water-soluble analgesic fenbufen (FBF) within cyclodextrin-based MOFs (CD-MOFs). The MOFs were immersed in an ethanolic solution of FBF for 24 h, achieving a significant loading efficiency of 196 mg/g. Another highly effective strategy for drug incorporation is post-synthetic modification (PSM), in which therapeutic molecules are attached to the MOF either on its surface or within its pore network after the initial framework has been synthesized. This process typically begins with the preparation of well-defined MOF nanoparticles possessing the desired size, morphology, and physicochemical stability. Drug incorporation into MOFs can occur via multiple types of interactions, including coordination with metal centers, covalent linkage to functional groups on the linkers, or adsorption on the external surface. Post-synthetic modification (PSM) allows precise regulation of both the distribution and the amount of drug loaded, thereby improving the efficiency of MOF-based drug-delivery systems [65,66]. For example, 5-fluorouracil (5-FU) was effectively loaded into ZIF-8 through a PSM method, achieving a remarkable loading capacity of around 660 mg of 5-FU per gram of ZIF-8 [67]. Covalent conjugation is another highly efficient strategy for incorporating drugs into MOFs. MOF surfaces are rich in reactive groups such as amino, carboxyl, and hydroxyl moieties, which can form stable covalent bonds with complementary sites on therapeutic molecules. These strong chemical connections enable secure and controlled encapsulation of active compounds within the MOF framework. Additionally, covalent attachment enhances the structural stability of the loaded molecules and allows fine-tuning of their release kinetics, supporting sustained delivery over prolonged periods. Applying covalent bonding in MOF functionalization thus represents a significant advancement, ensuring durable drug incorporation while addressing the limitations associated with non-covalent loading strategies [52,68].

As an illustration, Morris et al. [69] synthesized UiO-66-N_3_ nanostructures and employed a strain-promoted click reaction to covalently attach oligonucleotides onto the framework’s surface. This covalent functionalization minimized drug leaching and improved the stability of the anchored molecules. After encapsulating therapeutic agents, a thorough understanding of the drug-release mechanisms is essential for achieving effective delivery. Drug liberation from MOFs can proceed via multiple pathways, enabling precise control and targeted release. Among these, diffusion-controlled release is one of the most commonly applied mechanisms, where drug molecules migrate from the MOF pores along a concentration gradient. The release rate in such systems can be influenced by several factors, including pore dimensions, surface chemistry, and the strength of interactions between the drug and the MOF scaffold [59,70]. Additionally, drug release can occur through MOF degradation induced by physiological factors, including changes in pH, redox conditions, or enzymatic activity [59,71]. MOFs can also be designed to respond to specific stimuli, enabling controlled release of their cargo in reaction to environmental triggers such as pH, temperature, light, or magnetic fields. For example, pH-sensitive MOFs exhibit faster drug release under acidic conditions, making them especially suitable for targeted cancer therapies.

Lin et al. [72] developed a biocompatible MOF, Zn-GA, composed of zinc ions and L-glutamic acid (GA), which exhibited dual pH- and temperature-responsive drug release. The MOF achieved a methotrexate loading capacity of 12.85 wt.% at 80 °C. Under acidic conditions (pH 5.0), about 43% of the drug was released, increasing to 68% at higher temperatures, demonstrating both pH-sensitive and thermally enhanced release behavior. Drug release can also occur via cleavage of covalent bonds between the drug and MOF, such as acid–labile linkages that hydrolyze under acidic conditions. Similarly, Sun et al. [67] loaded 5-FU into ZIF-8, attaining a high loading of approximately 60%. The system showed controlled, pH-responsive release, with faster drug liberation at pH 5.0 than at pH 7.4—over 45% of 5-FU was released in the first hour under acidic conditions, compared to only 17% at neutral pH. Another release pathway involves structural degradation of the MOF under specific environmental stimuli, including pH changes or competing ions that disrupt metal–ligand coordination, leading to accelerated drug release [71].

Zhang et al. [73] engineered a pH-responsive MOF, MIL-101-NH_2_, designed for the co-delivery of curcumin and small interfering RNA (siRNA) targeting hypoxia-inducible factor 2α (HIF-2α). Both therapeutic agents were incorporated into the framework via encapsulation and surface coordination interactions. MIL-101-NH_2_ successfully protected siRNA from nuclease-mediated degradation and facilitated its escape from lysosomes. In acidic environments, the MOF underwent pH-triggered degradation, enabling the gradual release of encapsulated curcumin. The overall drug-release behavior is determined by the MOF’s structural design, the physicochemical properties of the loaded agents, and the intended release kinetics necessary to achieve therapeutic efficacy. For example, pH-sensitive MOF nanocarriers have been shown to release drugs more rapidly under acidic conditions, making them particularly suitable for targeted delivery to tumor tissues. The tunable nature of MOFs allows for precise control over release profiles, highlighting their potential as versatile platforms for advanced biomedical applications.

## 4. Curcumin-Loaded Nanoparticles for Drug Delivery and Cancer Therapy

Curcumin-loaded nanoparticles have emerged as highly effective drug-delivery systems (DDSs) capable of overcoming intrinsic limitations of curcumin, such as poor bioavailability and rapid metabolism. Various nanoscale platforms have been developed, including mesoporous silica nanoparticles (MSNs), niosomes, chitosan (CS) nanoparticles, and solid lipid nanoparticles (SLNs), offering advantages such as enhanced drug-loading capacity, controlled release, improved stability, and increased cellular uptake [25,74]. Some curcumin-based nanocarriers exhibit multifunctional properties; for example, self-fluorescent MSNs coated with a curcumin polymer shell can serve as imaging agents while improving drug loading and enabling stimuli-responsive release [75]. Similarly, curcumin-loaded ovalbumin nanoparticles demonstrate reverse-targeting functionality and provide controlled delivery for allergy therapy [76]. These multifunctional nanosystems exemplify the dual therapeutic and diagnostic potential of curcumin-loaded nanoparticles [74].

Nanoscale DDSs improve the solubility and bioavailability of curcumin, enabling tumor-targeted delivery with controlled release. Systems such as polymeric micelles, MSNs, and self-assembled peptide nanofibers enhance curcumin stability in aqueous media, thereby boosting therapeutic efficacy [77]. Targeted delivery is further facilitated via the enhanced permeability and retention (EPR) effect and through active targeting using tumor-specific ligands, including hyaluronic acid (HA), folic acid, and arginine–glycine–aspartic acid (RGD) peptide motifs, which increase tumor specificity and reduce off-target effects [78,79]. Moreover, stimuli-responsive features allow for controlled and sustained release at the tumor site, maximizing therapeutic outcomes while minimizing systemic toxicity [78].

Although a wide range of nanoparticle platforms have been explored to enhance the pharmacological properties of curcumin, systematic comparative studies are essential to guide their rational selection for specific clinical applications. Different nanocarrier formulations have demonstrated substantial improvements in curcumin’s solubility, bioavailability, and pharmacokinetics, as supported by quantitative evidence. For example, poly(lactic-co-glycolic acid) (PLGA) nanoparticles increased the aqueous solubility of curcumin by more than 11,000-fold, while liposomal formulations enhanced its oral bioavailability by approximately 9.5-fold compared with free curcumin [80]. Curcumin-loaded solid lipid nanoparticles (SLNs) further showed a 6.2-fold extension of plasma half-life and a 4.8-fold increase in cellular uptake relative to native curcumin [81]. These findings highlight the ability of nanocarriers to address pharmacokinetic limitations and their strong potential for clinical translation. However, the magnitude of these improvements depends on the type of nanomaterial, formulation parameters, and administration route, underscoring the need for systematic optimization in future studies.

## 5. MOFs for Delivery of Curcumin Against Cancer

Accumulating evidence demonstrates that curcumin-loaded MOFs can effectively promote apoptosis, inhibit cancer cell proliferation, and reduce tumor growth both in vitro and in vivo. The following studies illustrate notable progress in the development of MOF-based delivery systems for curcumin across various cancer types, highlighting their potential as versatile and promising platforms for next-generation anticancer nanomedicine. In comparison with conventional nanoscale formulations, MOF-based carriers offer significantly higher curcumin loading capacities, improved protection against hydrolytic degradation, and customizable release profiles owing to their tunable porosity and metal–linker chemistry. Among the various platforms investigated, zinc-based MOFs (such as ZIF-8 and bio-MOF derivatives), iron-based frameworks including MIL-101(Fe), and functionalized zirconium-based MOFs (for instance, PEGylated or aptamer-modified UiO frameworks) have emerged as particularly effective for curcumin delivery. These systems consistently exhibit high encapsulation efficiency, favorable biocompatibility, and either pH-responsive or sustained release characteristics. As summarized in Table 1, curcumin-loaded MOFs have demonstrated enhanced anticancer activity against diverse tumor models, including breast (MCF-7), gastric (AGS), colorectal (HT-29), and cervical (HeLa) cancer cells, largely due to improved cellular uptake and increased cytotoxicity compared with free curcumin. For the purpose of direct comparison, Table 2 provides a summary of key performance metrics for major MOF platforms employed in curcumin delivery, including drug-loading efficiency, release behavior, half-maximal inhibitory concentration (IC_50_), targeting strategies, and reported biological outcomes.

Moradi et al. [82] developed HA-targeted MOF nanoparticles based on NH_2_-MIL-101(Fe), namely platinum–curcumin@MIL@HA, for the delivery of the platinum-curcumin cytotoxic agent to MDA-MB-231 triple-negative breast cancer cells. In vitro cytotoxicity studies demonstrated that the platinum–curcumin-loaded MOF nanoparticles exhibited significantly greater cytotoxic effects compared with the free drug. Furthermore, cellular uptake experiments indicated that HA-mediated targeting substantially enhanced nanoparticle internalization relative to non-targeted counterparts.

Laha et al. [83] encapsulated curcumin within monodispersed, isoreticular nanoscale MOF nanoparticles (NMOF-3) to achieve targeted delivery against triple-negative breast cancer (TNBC) cells. The cytotoxicity of IRMOF-3, curcumin-loaded IRMOF-3 (IRMOF-3@curcumin), and folic acid (FA)-conjugated curcumin-loaded IRMOF-3 (IRMOF-3@curcumin@FA) was evaluated in both human and murine TNBC models. The FA-targeted system (IRMOF-3@curcumin@FA) induced apoptosis in human TNBC cells by upregulating the pro-apoptotic protein Bax, downregulating the anti-apoptotic protein Bcl-2, and activating key signaling mediators, including c-Jun N-terminal kinase (JNK) and p53. In vivo studies further demonstrated that mice receiving the targeted curcumin delivery system showed improved survival and a marked reduction in tumor volume compared with those treated with the non-targeted nanocarrier.

In a separate study, Alavijeh and colleagues [48] synthesized two zinc-based MOFs, DMOF-1 and NO_2_-functionalized DMOF-1 (DMOF-1-NO_2_), and used them to encapsulate curcumin for drug-delivery applications. Encapsulation within these MOF frameworks enhanced curcumin’s aqueous solubility and significantly increased its cytotoxicity against human gastric cancer (AGS) cells relative to free curcumin. These findings indicate that even MOFs with limited hydrolytic stability can serve as effective carriers to improve the bioavailability and anticancer efficacy of curcumin.

Nabipour and Hu [53] engineered a pectin-coated curcumin@bio-MOF-11 drug-delivery system to improve both the stability and therapeutic efficacy of curcumin. Cytotoxicity assays conducted on the human colon carcinoma cell line (SW489) indicated that the pectin/curcumin@bio-MOF-11 composite displayed markedly greater antitumor activity compared with free curcumin. In vitro release studies under simulated gastrointestinal conditions revealed a controlled and sustained release profile, highlighting the protective effect of the pectin coating and its responsiveness to colonic pH. Furthermore, MTT assays confirmed the pronounced cytotoxic effects of the composite on human colon carcinoma cells. Overall, these results demonstrate that bio-MOF-11 functions as a biocompatible and effective nanocarrier for targeted colonic delivery of curcumin, underscoring its strong potential for applications in colorectal cancer therapy.

Bazzazan et al. [84] synthesized and characterized UIO-66 MOFs as potential nanocarriers for the delivery of curcumin and assessed their anticancer activity against breast cancer cell lines. The resulting UIO-66-curcumin formulation exhibited significantly higher antitumor effects compared with either curcumin or UIO-66 alone. Encapsulation of curcumin within the UIO-66 framework substantially enhanced its efficacy, as evidenced by a markedly lower IC_50_ relative to free curcumin. Moreover, UIO-66-curcumin promoted apoptosis by upregulating caspase-3 and caspase-9 and simultaneously downregulated proteins associated with metastasis and cell proliferation, including matrix metalloproteinases (MMP-2 and MMP-9) and cyclins D and E. These results suggest that UIO-66 functions as an effective nanocarrier, improving the anticancer potential of curcumin by inducing apoptosis and inhibiting tumor progression.

Nabipour and colleagues [47] designed a novel bio-Schiff base ligand derived from vanillin to construct a copper-based bio-MOF. Curcumin was successfully incorporated into this framework, forming curcumin@bio-MOF, with an entrapment efficiency of approximately 84.35% and a loading capacity of 28.11%, demonstrating effective drug incorporation. To achieve controlled and tumor-specific release, the curcumin@bio-MOF was further encapsulated within a carboxymethyl cellulose (CMC) matrix, resulting in a pH-responsive CMC/curcumin@bio-MOF hydrogel. In vitro experiments revealed that the CMC coating significantly enhanced the sustained release of curcumin compared to the uncoated MOF. Cytotoxicity assays confirmed improved anticancer efficacy, and confocal laser scanning microscopy demonstrated efficient cellular uptake and targeted intracellular delivery. Overall, these findings highlight the CMC/curcumin@bio-MOF hydrogel as a biocompatible, pH-sensitive nanoplatform capable of precise tumor-targeted curcumin delivery with controlled release kinetics.

In a separate study, Nabipour et al. [54] prepared a zinc-based MOF (Zn-MOF) through the reaction of zinc acetate dihydrate with a Schiff base ligand. Curcumin was then encapsulated within the Zn-MOF, and the resulting nanocarrier was coated with the biopolymer sodium alginate (SA) to enable controlled and sustained release in specific environments. In vitro release experiments demonstrated that curcumin@MOF exhibited a pH-dependent release profile, with 78.9% and 50.0% of curcumin released at pH 5.0 and 7.4, respectively. The SA-coated curcumin@Zn-MOF showed superior release behavior compared with the uncoated MOF. Furthermore, in vitro cytotoxicity studies indicated potent antitumor activity, as the sustained release of curcumin from the MOF induced apoptosis and cell death in HeLa, HEK293, and SH-SY5Y cell lines.

In a recent study, Babaei et al. [85] designed and characterized bio-MOF nanoparticles employing curcumin as a ligand and iron as active centers to create a biocompatible and biodegradable drug-delivery system. The system was loaded with DOX, coated with PEG, and functionalized with an EpCAM aptamer to achieve colorectal cancer–specific targeting. In vitro experiments demonstrated that Apt-PEG-MOF@DOX effectively delivered DOX and exhibited pronounced cytotoxicity against HT-29 colorectal cancer cells, while showing minimal effects on normal cells. Furthermore, in vivo studies using immunocompromised C57BL/6 mice bearing HT-29 tumors revealed significant tumor growth inhibition, highlighting the strong therapeutic potential of the Apt-PEG-MOF@DOX nanocarrier.

Guo et al. [86] engineered a copper-based MOF-199 nanoplatform (curcumin@DOX@MOF-199) designed to co-deliver curcumin and DOX for inducing ferroptosis and apoptosis in MCF-7 human breast cancer cells. The study revealed that curcumin@DOX@MOF-199 activated caspase-3 and triggered chromatin condensation, confirming apoptotic cell death. In vitro analyses demonstrated significantly higher cytotoxicity and enhanced cellular uptake of the co-loaded MOF compared to free drugs or single-drug MOF formulations. Additionally, in vivo experiments in MCF-7 tumor-bearing mice showed substantial tumor growth inhibition with negligible systemic toxicity. Overall, these results suggest that curcumin@DOX@MOF-199 provides an effective nanoplatform for synergistic ferroptosis–apoptosis–based cancer therapy.

Nabipour et al. [87] recently fabricated a curcumin-loaded MOF, UWO-2 (curcumin@UWO-2), and subsequently developed a biocomposite, CS-κ-Cr/curcumin@UWO-2, by embedding the MOF within a chitosan (CS) and κ-carrageenan (κ-Cr) matrix. This nanoplatform was designed to enhance curcumin bioavailability, reduce cytotoxicity, and improve overall therapeutic efficacy. The material exhibited increased structural stability and a high surface area, facilitating efficient curcumin encapsulation and controlled release. Cytotoxicity studies indicated augmented cancer cell death mediated by curcumin@UWO-2, owing to the synergistic effects of the MOF framework, while the CS-κ-Cr coating provided sustained and regulated curcumin delivery. Confocal microscopy confirmed effective cellular uptake, highlighting the potential of this biocomposite for targeted cancer chemotherapy.

## 6. Conclusions and Future Directions

Curcumin-loaded nanoscale MOFs summarized in this review indicate meaningful improvements in drug loading, stability, and anticancer activity compared with free curcumin. Among the various MOF platforms evaluated in the reviewed studies, several formulations achieved high encapsulation efficiency and enhanced cytotoxicity in vitro, particularly against breast (MCF-7), gastric (AGS), colorectal (HT-29), and cervical (HeLa) cancer cells. These findings support the potential of MOFs as effective carriers for curcumin delivery in cancer therapy.

Although these results are promising, they also reveal specific limitations that need to be addressed to advance MOF-based curcumin delivery systems. Some MOFs exhibit hydrolytic sensitivity, potential metal ion release, or interactions with plasma proteins, all of which may influence biocompatibility and therapeutic performance. Polymer-coated, bio-MOF, and ligand-functionalized formulations have shown improved colloidal stability, reduced nonspecific interactions, and enhanced cellular uptake, suggesting that surface modification is a key strategy for optimizing these platforms.

Curcumin, like many polyphenols, demonstrates dose-dependent biological effects. At lower concentrations, it exhibits antioxidant and anticancer activities, while at higher levels, it may induce pro-oxidant or cytotoxic effects. Studies indicate that curcumin is well tolerated within typical therapeutic ranges, and toxicity is generally observed only at substantially higher exposures [11,88]. Therefore, future research should focus on detailed in vivo pharmacokinetic and toxicological evaluations to ensure biocompatibility and long-term safety.

Considering the favorable results demonstrated by the reviewed MOF systems, future work should also prioritize investigating how MOF composition, porosity, degradation behavior, and surface chemistry influence therapeutic outcomes across different cancer types. Overall, the evidence presented in this review underscores the strong potential of curcumin-loaded MOFs while emphasizing the need for thoughtful material optimization and rigorous biological evaluation to support their eventual clinical translation.

## Figures and Tables

**Table 1:** Summary of representative studies on curcumin-loaded MOFs for cancer therapy.

Study (Ref.)	MOF Type/Composition	Target Cancer/Cell Line	Functional Modification/Coating	Key Findings
Moradi et al. [82]	NH_2_-MIL-101(Fe)-based (Pt–Curcumin@MIL@HA)	Triple-negative breast cancer (MDA-MB-231)	HA targeting	Enhanced cytotoxicity vs. free drug; HA improved uptake and tumor selectivity
Laha et al. [83]	IRMOF-3 and FA-conjugated IRMOF-3@Curcumin	Triple-negative breast cancer (TNBC)	Folic acid targeting	Induced apoptosis via Bax/Bcl-2 modulation and p53 activation; reduced tumor volume
Alavijeh et al. [48]	Zn-based DMOF-1 and DMOF-1–NO_2_	Gastric cancer (AGS)	None	Improved solubility and enhanced cytotoxicity vs. free curcumin
Nabipour and Hu [53]	Pectin-coated Bio-MOF-11 (Curcumin@Bio-MOF-11)	Colon carcinoma (SW489)	Pectin coating	Controlled, pH-responsive release; superior cytotoxicity and colonic targeting
Bazzazan et al. [84]	UIO-66–Curcumin	Breast cancer	None	Enhanced apoptosis via caspase-3/9 activation; reduced IC_50_; downregulated MMP-2/9 and cyclins D/E
Nabipour et al. [47]	Bio-Schiff base Cu–MOF (Curcumin@Bio-MOF)	Cervical (HeLa)	CMC coating	pH-sensitive hydrogel achieved sustained release and enhanced anticancer activity
Nabipour et al. [54]	Zn–MOF with Schiff base ligand	HeLa, HEK293, SH-SY5Y	SA coating	Controlled pH-dependent release (78.9% at pH 5.0); strong anticancer activity with sustained release
Babaei et al. [85]	Bio-MOF (Fe-based, PEGylated, Aptamer-functionalized)	Colorectal cancer (HT-29)	PEG + EpCAM aptamer + DOX co-loading	High tumor-specific cytotoxicity; significant tumor inhibition in mice
Guo et al. [86]	Cu–MOF-199 (Curcumin@DOX@MOF-199)	Breast cancer (MCF-7)	DOX co-loading	Induced apoptosis and ferroptosis; strong in vivo tumor suppression with low toxicity
Nabipour et al. [87]	Curcumin@UWO-2 and CS–κ-Cr/Curcumin@UWO-2	General cancer models	κ-carrageenan + chitosan composite	Enhanced stability, sustained release, and improved cytotoxicity

Abbreviations: MOF: Metal–organic framework; MIL-101(Fe): iron-based MIL-101 MOF; Pt: platinum (nanoparticles); HA: hyaluronic acid; FA: folic acid; IRMOF-3: isoreticular MOF-3; AGS: human gastric adenocarcinoma cell line; UiO-66: zirconium-terephthalate MOF; CMC: carboxymethyl cellulose; SA: sodium alginate; PEG: polyethylene glycol; DOX: doxorubicin; EpCAM: epithelial cell adhesion molecule; IC_50_: half-maximal inhibitory concentration; MMP-2/9: matrix metalloproteinases-2/9; HeLa: human cervical cancer cell line; HEK293: human embryonic kidney 293 cell line; SH-SY5Y: human neuroblastoma cell line; HT-29: human colorectal adenocarcinoma cell line; MCF-7: human breast adenocarcinoma cell line; CS: chitosan. κ-Cr, κ-carrageenan.

**Table 2:** Comparison of curcumin-loaded MOFs.

MOF System (Ref.)	Drug Loading/Entrapment Efficiency	Release Behavior	IC_50_/Cytotoxicity Performance	Targeting/Functional Features
NH_2_-MIL-101(Fe)-based (Pt–Curcumin@MIL@HA [82]	Not reported	Sustained release; enhanced uptake under acidic tumor-like conditions	Higher cytotoxicity than free drug in MDA-MB-231 triple-negative breast cancer cells	HA enables receptor-mediated active targeting
IRMOF-3@Curcumin@FA [83]	Not reported	Controlled release	Strong apoptosis; improved survival in triple-negative breast cancer (TNBC) mouse model	FA enhances tumor selectivity
DMOF-1/DMOF-1-NO_2_ (Zn-based) [48]	Improved encapsulation; NO_2_ derivative shows superior loading	Not reported	Higher cytotoxicity vs. free curcumin in AGS cells	NO_2_ functional group strengthens interactions
Pectin-Coated Curcumin@Bio-MOF-11 [53]	EE ≈ 84.35%	Sustained, pH-responsive colonic release	Higher cytotoxicity vs. free curcumin in SW489 cells	Pectin coating ensures colon-specific release
UIO-66@Curcumin [84]	Not reported	Stable, controlled release	Lower IC_50_; caspase-3/9 activation; MMP-2/9 inhibition	Stable zirconium-MOF enhances apoptosis and suppresses metastasis
CMC/Curcumin@Bio-MOF (Cu-based) [47]	EE ≈ 84.35%; Loading ≈ 28%	pH-dependent, sustained release	Enhanced cytotoxicity and cellular uptake	CMC improves biocompatibility & pH responsiveness
SA-Coated Curcumin@Zn-MOF [54]	Not reported	pH-responsive release: 78.9% (pH 5) vs. 50% (pH 7.4)	Apoptosis in HeLa, HEK293, SH-SY5Y cells	SA enhances stability and controlled delivery
Apt-PEG-MOF@DOX (Curcumin-Ligand Bio-MOF) [85]	DOX-loaded system	Controlled release	Selective cytotoxicity toward HT-29 cells; low toxicity in normal cells	EpCAM aptamer provides cancer-specific targeting
Curcumin@DOX@MOF-199 (Cu-based) [86]	Co-loading of DOX + curcumin	Sustained dual-drug release	Synergistic ferroptosis + apoptosis; strong tumor inhibition	Dual-drug therapeutic nanoplatform
Curcumin@UWO-2/CS-κ-Cr/Curcumin@UWO-2 [87]	High loading; increased with polymer	Controlled release; reduced burst with CS/κ-Cr coating	Strong cytotoxicity; improved safety profile	Biocomposite improves biocompatibility and sustained release

Abbreviations: MOF: Metal–organic framework; MIL-101(Fe): iron-based MIL-101 MOF; Pt: platinum (nanoparticles); HA: hyaluronic acid; FA: folic acid; IRMOF-3: isoreticular MOF-3; AGS: human gastric adenocarcinoma cell line; W489: Human colon carcinoma cell line; UiO-66: zirconium-based MOF; IC_50_: half-maximal inhibitory concentration; MMP-2/9: matrix metalloproteinases-2/9; CMC: carboxymethyl cellulose; SA: sodium alginate; HeLa: human cervical cancer cell line; HEK293: human embryonic kidney 293 cell line; SH-SY5Y: human neuroblastoma cell line; PEG: polyethylene glycol; DOX: doxorubicin; HT-29: human colorectal adenocarcinoma cell line; EpCAM: epithelial cell adhesion molecule; CS: chitosan; κ-Cr, κ-carrageenan.

## Data Availability

No new data were created or analyzed in this study.

## Abbreviations

AlMOF	Aluminum fumarate metal–organic framework
BET	Brunauer–Emmett–Teller
CD-MOFs	Cyclodextrin-based metal–organic frameworks
CMC	Carboxymethyl cellulose
DDSs	Drug-delivery systems
DOX	Doxorubicin
EPR	Enhanced permeability and retention
FA	Folic acid
FBF	Fenbufen
GA	L-glutamic acid
HIF-2α	Hypoxia-inducible factor 2α
IBU	Ibuprofen
IC_50_	Half-maximal inhibitory concentration
ICP-MS	Inductively coupled plasma mass spectrometry
IR	Infrared radiation
JNK	c-Jun N-terminal kinase
κ-Cr	κ-carrageenan
MAPK	Mitogen-activated protein kinase
MOFs	Metal–organic frameworks
MMP	Matrix metalloproteinase
MSNs	Mesoporous silica nanoparticles
NCDs	Noncommunicable diseases
NF-κB	Nuclear factor kappa B
NMR	Nuclear magnetic resonance
PI3K/Akt	Phosphoinositide 3-kinase/protein kinase B
PLGA	Poly (lactic-co-glycolic acid)
PSM	Post-synthetic modification
PXRD	Powder X-ray diffraction
RGD	Arginine–glycine–aspartic acid
ROS	Reactive oxygen species
SA	Sodium alginate
SCXRD	Single-crystal X-ray diffraction
SEM	Scanning electron microscopy
siRNA	Small interfering RNA
SLNs	Solid lipid nanoparticles
STAT3	Signal transducer and activator of transcription 3
TEM	Transmission electron microscopy
TGA	Thermogravimetric analysis
TNBC	Triple-negative breast cancer
XPS	X-ray photoelectron spectroscopy
ZIF-8	Zeolitic imidazolate framework-8
